# Optical Investigation of DNA Behaviour in Flow using Confocal Detection Incorporated with Fluorescence Correlation Spectroscopy (FCS)

**DOI:** 10.1007/s10895-025-04263-5

**Published:** 2025-03-20

**Authors:** Fatma Doğan Güzel, Hamed Ghorbanpoor

**Affiliations:** 1 Department of Mechanical Engineering, Faculty of Engineering and Natural Science, Ankara Yıldırım Beyazıt University, Ankara, Turkey; 2 Department of Chemistry, Imperial College London, London, UK; 3 Department of Biomedical Engineering, Eskisehir Osmangazi University, Eskisehir, Turkey

**Keywords:** DNA behaviour in Flow, Microfluidics, Rheological properties of DNA, Fluorescence Correlation Spectroscopy (FCS), Flow-dominated regime

## Abstract

Investigation of the behavior of biopolymers in flow such as Deoxyribonucleic acid (DNA), is a critical challenge in engineering, polymer, and life sciences. In this study, we studied the rheological properties of DNA and flow characteristics in real-time. The velocity measurement was carried out using confocal detection incorporated with Fluorescence Correlation Spectroscopy (FCS). Optical experiments provided an understanding of the diffusion- and flow-dominated regimes for molecules treated in microfluidic channels and lab-on-chip devices in general. We found that the flow-dominated regime starts at a flow rate of 0.3 µl/min and the transitional regime falls into 0.02–0.3 µl/min flow rates. There are a few examples for the detection of DNA and different fragments in flow as such. It is therefore believed to provide valuable insights into the subject of flow dynamics of DNA.

## Introduction

The behavior of flow-induced particle migration has been studied via various techniques for almost a century [1, 2]. Early studies include the use of Pitot-static tubes and hot-wire anemometers; however, both are invasive methods which require the insertion of a physical probe into the flow stream. With the invention of the laser, a non-invasive method was introduced; laser-probed Doppler anemometer, but this requires sophisticated electronics and optics making it an undesirable choice for velocity measurements. Other non-invasive techniques based on instantaneous optical particle tracking have been developed. For example, particle image velocimetry (PIV) is considered a technique that requires sophisticated equipment. It works by capturing sequential image frames of the particles illuminated by a laser sheet and then using cross-correlation algorithms to calculate the displacement of particles between frames, which determines the velocity field [3]. Recent advances in optical imaging have paved the way for single-molecule detection in flowing samples with increased sensitivity and reduced detection volumes such as laser-induced fluorescence reported by Keller et al. [4, 5]. In a similar manner, the combination of confocal detection with fluorescence correlation spectroscopy (FCS) has been employed to perform highly sensitive flow velocity measurements based on single-molecule detection [6]. FCS was first introduced in 1970’s [7, 8]. However applications of FCS in flowing systems was first shown theoretically and then experimentally by Magde et al. in 1978 [9]. FCS is now a well-established technique that enables the study of reaction kinetics and dynamics related to rotational diffusion, translational diffusion and flow velocities of molecules. The idea is that when fluorescently labelled molecules pass through a detection/probe volume defined by the laser beam, stochastic fluorescence bursts are detected and recorded over time. Burst analysis is performed by using photon counting histograms (PCH) where the distribution of the number of photons in each burst is defined statistically. FCS is employed for further statistical analysis of the bursts in order to understand the primary source of the concentration fluctuations. Its applications in microfluidic channels have been shown by various researchers only after the establishment of microfluidic devices [6, 10–12]. Deng and et al*.* proposed a novel strategy for studying drug-target protein interactions in single living cells by using a fluorescent probe-target complex and FCS with a microfluidic chip [13]. Cecchini et al*.* reported flow-based autocorrelation studies to detect and characterize single metallic nanoparticles using surface-enhanced resonance Raman spectroscopy. The benefits of flow-based systems over diffusion-limited approaches are highlighted, and a new model is proposed to fit the autocorrelation curve [12].

In a microfluidic system, DNA molecules can be manipulated and studied with high precision and control, allowing researchers to gain insights into the fundamental physics of DNA as well as its biological functions [1, 14]. One of the key advantages of microfluidics is the ability to handle small sample volumes, which reduces the cost of experiments and can enable high-throughput analysis. Microfluidic systems have found many applications in biological science such as pathogen detection, antibiotic susceptibility testing study of hybridization and DNA detection/sequencing and so on [15–17]. DNA studies in microfluidic systems are of particular interest due to the lack of understanding in the behavior of the molecule in static and hydrodynamic confined environments [18–20]. For instance, Hernández-Neuta et al., reported a microfluidic device that uses a magnetic fluidized bed to effectively analyze DNA. This device allows for continuous operation, which speeds up and improves the accuracy of capturing, separating, and detecting DNA. Because of its innovative design, this system outperforms conventional techniques and is well-suited for fast, automated genetic analysis. [19]. Wu et al., developed a microfluidic method to accurately align and stretch single DNA molecules on surfaces, allowing for high-throughput analysis. This technique creates a more uniform and parallel arrangement of DNA strands, which makes it easier to study genetic structures and molecular interactions at the single-molecule level in detail. Compared to standard DNA combing methods, this new approach offers better control, can be scaled up, and is more efficient, making it a useful tool for genomic research and diagnostic applications [21].

**Fig. 1:**
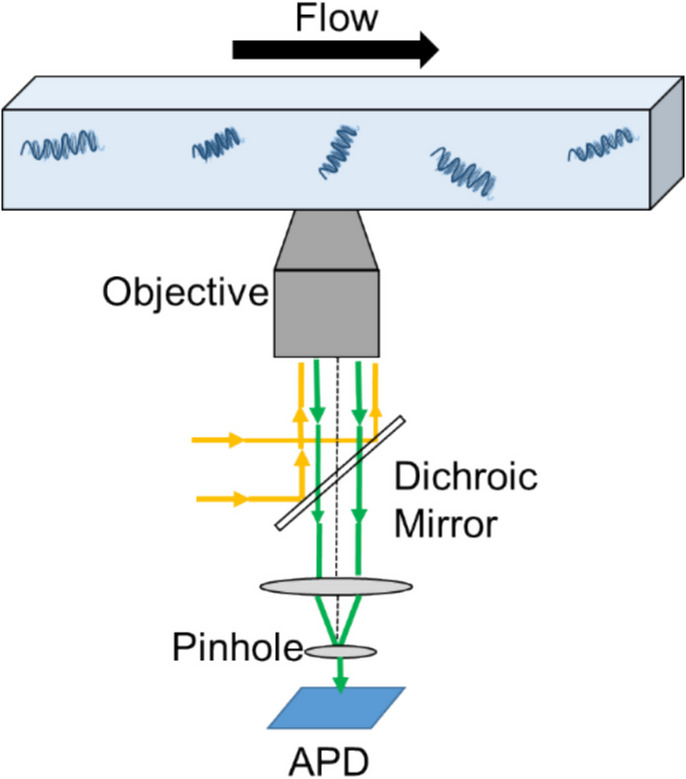
A diagrammatic representation of an FCS configuration that utilizes confocal configuration

In microfluidic systems, FCS can be used to study the behavior of DNA in confined spaces and under different flow conditions. For example, FCS has been used to measure the diffusion coefficient of DNA in microchannels with different geometries and surface properties [22]. FCS has also been used to study the binding kinetics of DNA with proteins or other molecules in microfluidic systems [23]. In this study, we introduce an innovative approach for studying rheological properties of DNA and flow characteristics in real-time and in their natural environment. The method combines FCS with a microfluidic chip to investigate the flow-dominated regime. Figure 1 demonstrates a schematic representation of the system.

## Experimental Section

### Optical Configuration

A custom-built confocal fluorescence microscopy (FM) set-up was used. This was built around a commercial microscope (Olympus IX71). The microscope is equipped with a mercury lamp and an Electron Multiplying Charge-Coupled Device (EMCCD) camera (Photometrics, The cascade II 1024) with a Quantum Efficiency (QE) of 90% for wide-field imaging. The camera is thermoelectrically cooled down to −60 ºC for maximum performance. A water immersion 60 × objective (UPlanSapo 60XW, Olympus) with a numerical aperture of 1.20 was used for imaging. Working distance of the objective is 280 µm. The image acquisition was performed with an open-source software package; Micro–Manager, which runs as a plugin to ImageJ [24]. The software settings were as follows; binning was 1, the exposure time and gain was kept constant at 0.1 ms and 3, respectively. Brightness and contrast were adjusted manually from the software. Confocal imaging was performed using a 488 nm continuous air-cooled argon ion laser (Coherent UK LTD, UK) and an avalanche photodiodes (APD) detector (Perkin-Elmer Optoelectronics, France) with green fluorescence emission in the spectral region.

### Device Preparation

A microfluidic poly (dimethylsiloxane) (PDMS) (Sylgard 184A, Dow and Corning, UK) chip with a channel dimension of 6 mm × 75 µm × 75 µm (length, width, depth, respectively) was fabricated by SU-8 master (MicroChem, UK) and bonded onto a 160 µm thick microscope cover slip [25, 26]. However, PDMS chips were specifically produced to have a thickness of about 120 um. Initially the surface of the PDMS chip and the cover slip were sonicated in ethanol for 15 min, dried with N_2_, and exposed to O_2_ plasma for about 30 s prior to bonding. The chip was then placed on a hot plate at 65 ºC to ensure strong bonding [26]. Upon the bonding, it was mounted onto the transitional stage of the microscope, the inlet of the channel connected to the syringe pump (Harvard Apparatus, UK), and the outlet to a vial. Finally, gas-tight syringes were applied in this study. The experiments were performed in shear flow.

### DNA Labelling with YOYO

YOYO −1 (1,1’-(4,4,7,7-tetramethyl-4,7-diazaundecamethylene)-bis-4-[3-methyl-2,3-dihydro-(benzo-1,3-oxazole)−2-methylidene]-quinolinium tetraiodide) intercalates with dsDNA and upon binding a strong fluorescence enhancement is observed on account of its high association constant [27, 28]. The absorption and emission wavelengths of YOYO-1/DNA complex are 491 nm and 509 nm respectively and are within the detection capability of the filters used. In this study, λ-DNA (New England BioLabs, UK) was stained with YOYO-1 at 5:1 base-pair:dye ratio. A 3 µl solution of 1 mM YOYO-1 iodide was mixed with 10 µl of 15.5 nM λ-DNA stock solution and incubated at room temperature for approximately 20 min. The mixture was diluted to 1 ml to give a concentration of 155 pM and 15.5 pM DNA/YOYO complex.

## Results and Discussion

FCS is best performed when Signal-to-Noise Ratio (SNR) is sufficiently high. There are couple of factors affecting SNR, including sample concentration, intensity changes in the laser light sources, quantum yield of the dye-molecule complex and detector efficiency [29, 30]. As for the sample concentration, one has to confirm that only a single molecule is present in the probe volume. For a 1 nM sample concentration the volume occupancy is 0.6 molecules per 1 fl volume. The number of fluorophores in the volume is described by the Poisson distribution, where the probability is around 0.33 for one, 0.10 for two molecules [31], approaching to 0.55 when the volume does not contain any molecule. In more dilute samples; SNR becomes highly sufficient for single molecule detection as the probability for two or more molecules in the volume drops to almost zero [32]. In order to determine the optimal working conditions for single molecule detection and higher SNR, 3 sets of experiments with 2 different concentrations of DNA and salt solution were carried out; 155 pM and 15.5 pM λ-DNA in 10 mM potassium chloride (KCl), and 15.5 pM λ-DNA in 1 M KCl. Flow rate was varied between 0 µl/min and 2 µl/min. The time traces were collected with 50 µs resolution and re-sampled with 200 µs resolution. Real-time autocorrelation curves were generated every 500 ms (N = 50,000) within a 60-s recording time using the normalized autocorrelation equation.

In FCS measurements, the technique evaluates the self-similarity of the fluctuations in a time scale by comparing the signal with itself at a time of t and after a delay time of τ. The normalized fluorescence correlation function can be written as (Eq. 1);

1 $$G\left(\tau\right)=\frac{\left\langle\delta I\left(t\right)\delta I\left(t+\tau\right)\right\rangle}{\left\langle I\left(t\right)\right\rangle^2}$$

where δI(t) and δI(t + τ) are the amplitudes of the fluctuations from the mean at time t and t + τ, respectively, and < I(t) > is the mean of the signal.

**Fig. 2:**
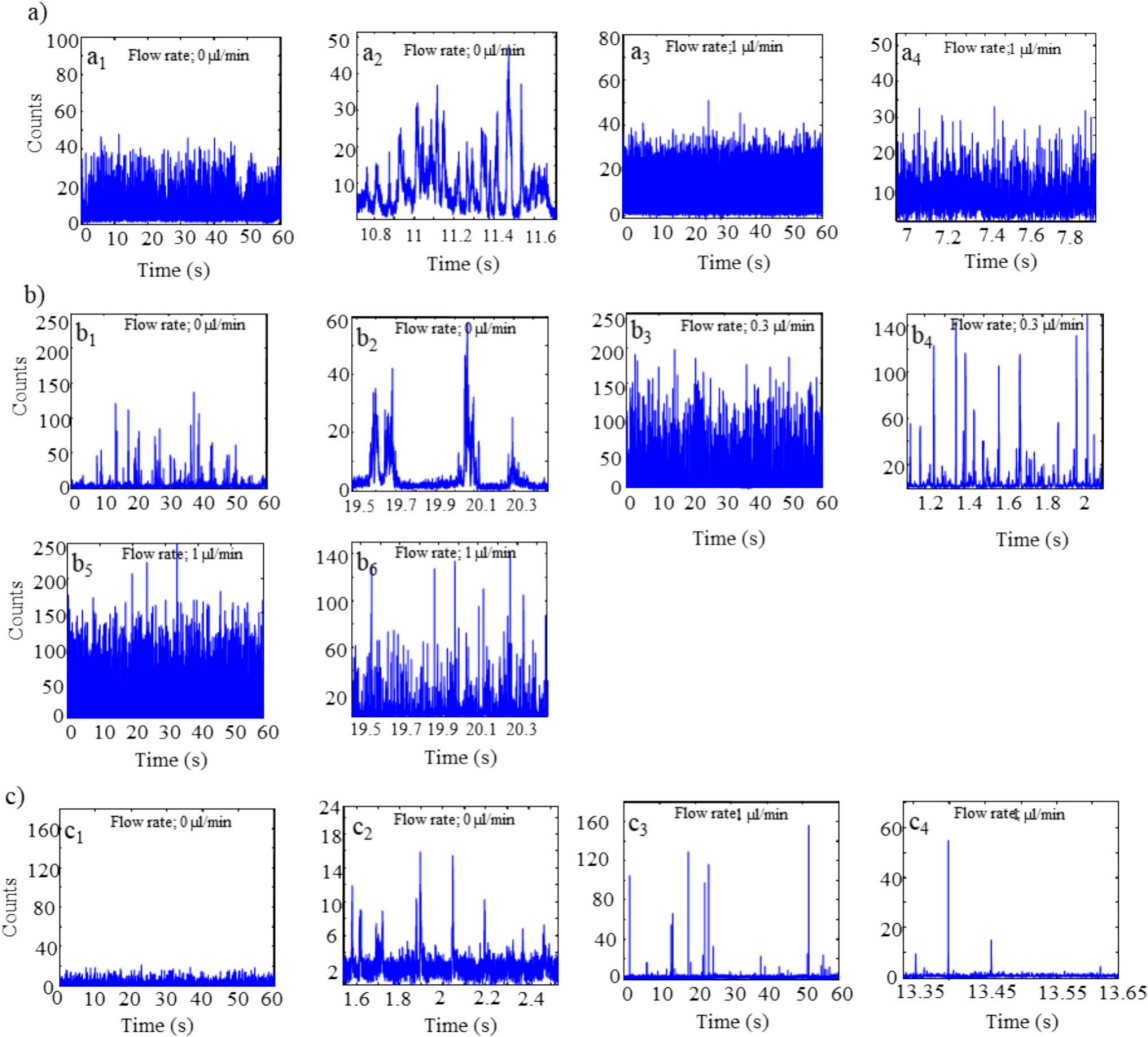
Representative single-molecule fluorescence burst scans of **a**)155 pM in 10 mM KCl (a_1_. 60 seconds time trace at 0 µl/min, a_2_. Expanded 1 second time trace of panel ‘a_1_’, a_3_. 60 seconds time trace at 1 µl/min, and a_4_. Expanded 1 second time trace of panel ‘a_3_’), **b**)15.5 pM λ-DNA in 10 mM KCl (b_1_. 60 seconds time trace at 0 µl/min. b_2_. Expanded 1 second time trace of panel ‘b_1_’. b_3_. 60 seconds time trace at 0.3 µl/min. b_4_. Expanded 1 second time trace of panel ‘b_3_’. b_5_. 60 seconds time trace at 1 µl/min. b_6_. Expanded 1 second time trace of panel ‘b_5_’.), and **c**)15.5 pM λ-DNA in 1 M KCl. (c_1_. 60 seconds time trace at 0 µl/min. c_2_. Expanded 2 seconds time trace of panel ‘c_1_’. c_3_. 60 seconds time trace at 1 µl/min. c_4_. Expanded 2 seconds time trace of panel ‘c_3_’.).

Figure 2a and b show several time traces of fluorescent burst scans recorded using 155 pM and 15.5 pM complex solution in 10 mM KCl at different flow rates (e.g. 0, 0.3, and 1 µl/min), respectively. The SNR increased significantly from almost 8 to 80 when a 10 × diluted complex solution was used. It is a consequence of the low volume occupancy. For a 1 fL volume, it is expected that the probe volume contains an average of 0.1 molecules for 155 pM and 0.01 for 15.5 pM, where the probability of detecting discrete events is higher.

The effect of salt concentration on the fluorescence signal intensity was also studied, as shown in Fig. 2c. For a 15.5 pM DNA solution, the SNR dropped to 5 at flow rate of 0 µl/min when using a 1M KCl buffer. At 1 µl/min, there was a significant decrease in the number of fluorescent bursts although SNR did not change significantly from that in 10mM KCl, as shown in Fig. 2b. Salt effect is a consequence of quenching of YOYO in high concentration of chloride ions.

**Fig. 3:**
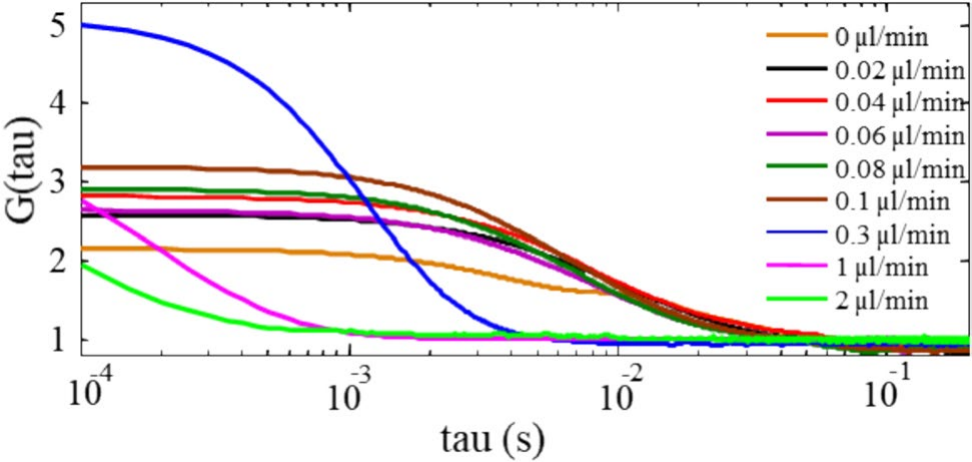
a) Autocorrelation curves of 15.5 pM sample solution for different flow rates

Therefore, FCS studies were focused on the data obtained with 15.5 pM sample concentration in 10 mM KCl. Figure 3 shows the autocorrelation curves obtained from the burst scans at flow rates between 0 µl/min and 2 µl/min. An immediate observation is that there is a clear shift in the Full Width Half Maxima (FWHM) from right to left in the time scale. It can also be seen that the amplitude of G(τ) increases with increasing flow rate. Unfortunately, the data collected at flow rates above 0.3 µl/min was truncated. Nonetheless, the data does not appear to contradict the trends just stated. FWHM calculated from the relevant fits to the autocorrelation curves corresponds to the related time scales such as τ_diff_ or τ_flow_, whereas G(τ) at τ = 0 represents the occupancy of the probe volume; namely the number of molecules occupying the probe volume at a time.

A variety of physical mechanisms might influence FCS. The principle contribution arises from diffusion with a time scale longer than 10^–5^ s. Other sources include triplet crossing, intermolecular processes, rotational diffusion, photobleaching and flow [33]. In order to reveal the time scales of contributing processes, the function has to be fit to a relevant theoretical model. For a system where diffusion dominates the fluctuations, a 3D *diffusion* model for the autocorrelation functions is given by Eq. 2;

2 $${G\left(\tau\right)}_{diff}=1+\left(\frac1N\right)A$$

where N is the mean number of particles in the probe volume and A (Eq. 3);

3 $$A=\left(1+\frac\tau{\tau_{diff}}\right)^{-1}\left(1+\frac\tau{\gamma^2\tau_{diff}}\right)^{-1/2}$$

where τ_diff_ is the average diffusion time (or correlation time) and Ɣ is called ‘*structure factor*’. τ_diff_ is given by (Eq. 4);

4 $${\tau }_{diff}=\frac{{w}_{0}^{2}}{4D}$$

and Ɣ is defined by (Eq. 5);

5 $$\gamma=\frac{w_0}{z_0}$$

where w_0_ is the laser beam waist (1/e^2^ radii of the laser beam), D is the translational diffusion coefficient and z_0_ is the probe depth.

In a flowing system, a theoretical 3 Dimension (3D) fit to the autocorrelation function is given by Eq. 6 [6, 9, 11];

6 $${G\left(\tau \right)}_{diff+flow}=1+\frac{1}{N}Aexp\left\{{(\frac{\tau }{{\tau }_{flow}})}^{2}A\right\}$$

where τ_flow_ is the average flow time of the molecules through the probe volume. By using τ_flow_ deduced from the experimental data, the flow velocity is calculated by Eq. 7;

7 $${V}_{flow}=\frac{{w}_{0}}{{\tau }_{flow}}$$

The physical meaning of the data can be studied quantitatively by fitting the curves with a relevant model, as described earlier. The autocorrelation curve obtained in the absence of flow was fitted with the 3D *diffusion* model, Eq. 3, and the others with the 3D *diffusion* + *flow* model, Eq. 6. The value for τ_diff_ deduced from the fit of the 3D *diffusion* model to the data collected under no flow was then fixed when fitting the data collected under flow and τ_flow_ determined for each flow rate. This approach has the advantage of providing a means of determining the diffusion- and flow-dominated regimes in a flowing system.

**Fig. 4:**
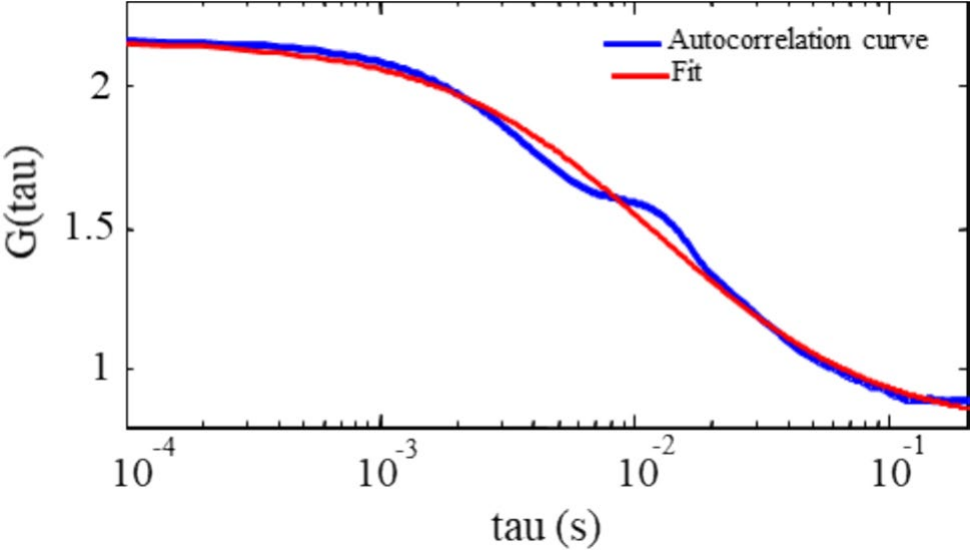
Autocorrelation curve and the fit for 0 µl/min flow rate (blue and red, respectively). The fitting function is the 3D diffusion model

Figure 4 shows the autocorrelation curve of 0 µl/min flow rate fitted by the 3D *diffusion* model, respectively. Although there seems to be a second unknown component in the autocorrelation curve, the curve could be fitted with less than 2% error. This second component might arise from the kinetics of conformational changes of DNA since DNA is a mobile structure, unlike a nanoparticle, that diffuses in and out of the probe volume with more fluctuations due to its chain flexibility [34]. Another possible reason for this might simply be due to the absorption of DNA complex onto the cover slip or the reflection of free-YOYO adsorption to the PDMS, which was experimentally observed by wide-field imaging.

The fit yields a τ_diff_ value of 12.5 ms. Using Eq. 4, the experimental diffusion coefficient of DNA (D_DNA_) can also be calculated and compared to literature for further validation.

**Fig. 5:**
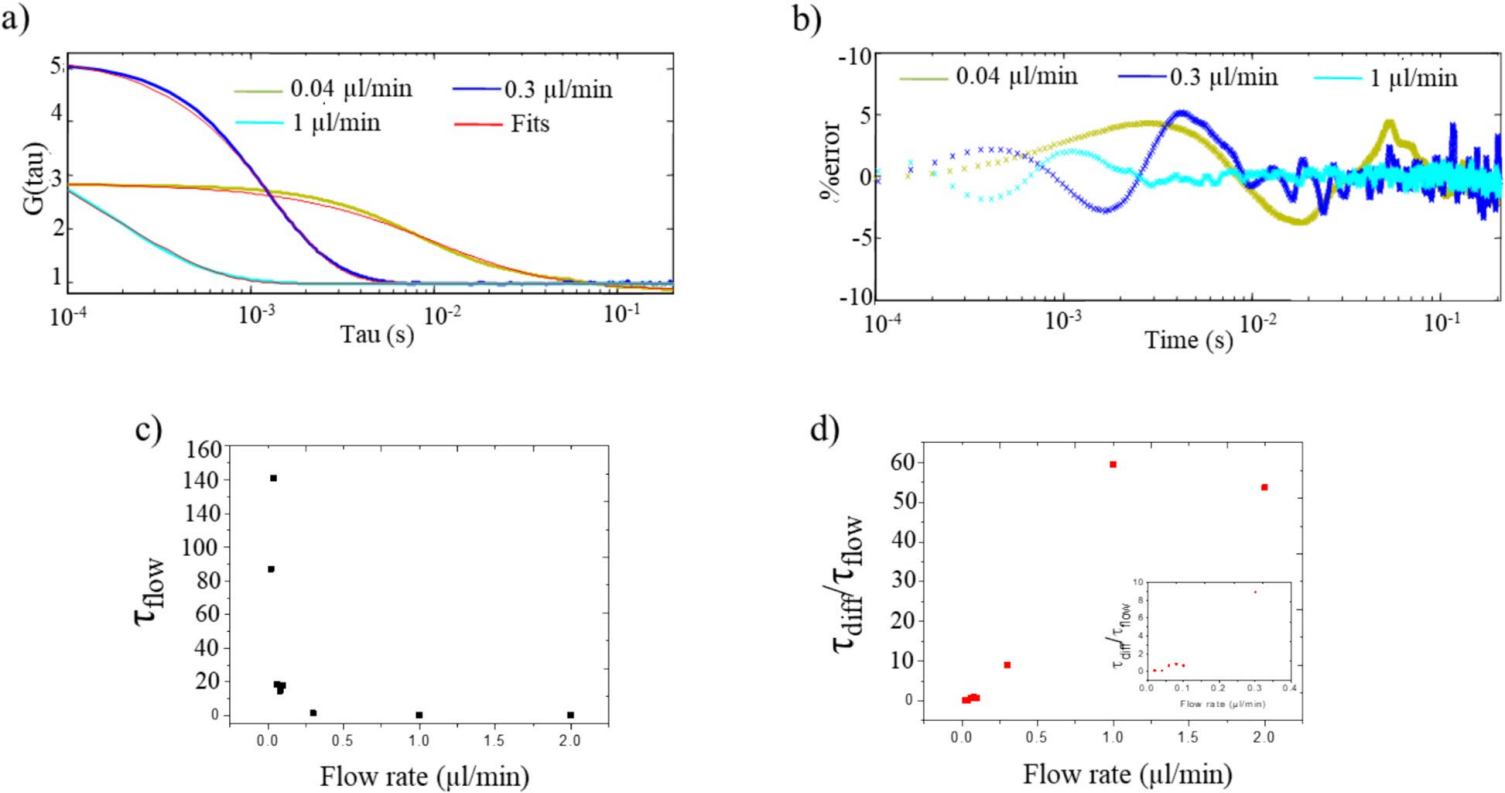
**a**) Autocorrelation curves and **b**) % error of 15.5 pM sample solution for selected flow rates. Flow rate versus τ: **c**) τ_flow_ versus flow rate and d) τ_diff_/τ_flow_ versus flow rate

To calculate w_0_, the autocorrelation curve of a dye with a well-known diffusion coefficient is investigated, and τ_diff_ and w_0_ are found accordingly, using Eqs. 2, 3 and 4. It was measured to be 568 nm by William Pitchford and this yields D_DNA_ to be 6.45 × 10^–12^ m^2^/s, which is in good agreement with the literature (Smith et al. reports that the self-diffusion coefficient of a single is 0.47 × 10^–12^ m^2^/s for λ-DNA [35]). Using the calculated τ_diff_, the 3D *diffusion* + *flow* model (Eq. 6) was fit to the autocorrelation functions obtained under different flow rates. Several examples along with the fits (i.e. 0.04 µl/min, 0.3 µl/min and 1 µl/min) and the relevant error analysis are shown in Fig. 5a. The second component that was observed at 0 µl/min flow rate is not discernable with the increased flow rate and each autocorrelation function was fit with less than 5% error (Fig. 5b).

The values of τ_flow_ extracted at each flow rate are plotted in Fig. 5c. An interesting observation is that τ_flow_ decreased more than 100 times with increasing flow rate before reaching a plateau at a flow rate of 0.3 µl/min where it was 10 times smaller in magnitude than τ_diff_. When τ_diff_ becomes greater than τ_flow_, the system is no longer diffusion-dominated, rather flow-dominated [12]. Therefore, the above result is an indication of the flow-dominated regime at 0.3 µl/min flow rate.

Investigating DNA behavior under flow is important because it can provide insights into the transport and deformation of DNA in microscale flows. The conformational dynamics and rheological properties of flexible polymers like DNA under various types of flow (e.g. uniform, extensional, shear, mixed) have been studied extensively [36–38]. It is well-known that if there is no external force impinging on the molecule, the molecule stays in its relaxed coiled state. However, in the presence of flow, the molecule undergoes several conformational distortions in its chain due to the force balance between Brownian or elastic coiling forces and stretching drag forces [39–41]. The stretching drag force resulting from flow velocity and the reducing hydrodynamic interactions as the molecule uncoils results in the molecule becoming elongated with increasing flow velocity. Table 1 compares the behavior and analysis of small and large DNA lengths in flow.

**Table 1:** Comparison of small and large DNA lengths, focusing on their behavior and analysis in flow

Aspect	Small DNA length	Large DNA length
Diffusion	Faster diffusion due to shorter contour length	Slower diffusion due to longer contour length and higher molecular weight
Relaxation Time	Shorter relaxation time, less prone to conformational changes	Longer relaxation time, more susceptible to stretching and coiling
Flow-Induced Elongation	Less sensitive to flow; minimal elongation even at higher shear rates	More sensitive to flow; significant elongation at lower shear rates
Conformational Dynamics	Simpler dynamics; less variability in end-to-end distances	Complex dynamics; broader distribution of end-to-end distances
FCS Analysis	Autocorrelation function primarily reflects diffusion and flow	Autocorrelation function includes contributions from conformational changes
Weissenberg Number (Wi)	Higher Wi required to induce elongation	Lower Wi required to induce elongation

The transition from a coiled to elongated state is expected to be different depending on the velocity gradient and flow type. In shear flow, the molecule experiences 2 steps of distortions as opposed to a sharp transition in extensional flow [42];

- For slow flow velocities**;** the relative distortions are proportional to the relaxation time of the molecule.
- For larger flow velocities; two states are possible depending on the magnitude of the flow rate. An intermediate state is observed at relatively lower flow velocity, where elongation is saturated as the molecule maintains a partially coiled state. At very high flow velocities, hydrodynamic interactions between DNA base-pairs are decreased sufficiently and the molecule becomes fully elongated.

The degree of the elongation can be determined by a dimensionless number called the ‘*Weissenberg number*’ (Wi). Wi is the product of the shear rate and the extensional relaxation time of the molecule in question, as illustrated in Eq. 8;

8 $$Wi=\dot{\gamma }\upxi$$

where $\dot{\gamma }$ is the shear rate and ξ is the longest relaxation time. The shear rate is a function of the velocity gradient and given by Eq. 9;

9 $$\dot{\gamma }=\frac{dV}{dx}$$

It is clear that the higher the Wi the higher the elongation because of increased shear rate in the function. Unfortunately, determining a precise Wi value for the transition from a coiled to elongated state is not trivial because of the complexity of the modelling.

In one theoretical study, Chen et al. reported that elongation is only expected when Wi >  > 1 [43]. At lower Wi, the flow would not create a sufficient force to deform the molecule from its relaxed state. With a modest approximation, Larson et al. suggested that the transition occurs at a critical value of Wi (0.275) in mixed shear and elongational flow [41]. These values differ due to the application of different flow and use of a different theoretical model. Smith et. al reported that the extension is much sharper in elongational flow compared to that in shear flow, and the probability distribution of extension in shear flow is very low at Wi < 2.5 [44]. For example, weak deformation from the coiled state in shear flow occurs at Wi = 1.3, $\dot{\gamma }$ = 0.2, corresponding to an extension of less than 5 µm for λ-DNA. Extension increases up to half of DNA contour length with a standard deviation of approximately 4 µm when Wi is increased to 76, $\dot{\gamma }$ = 4.0. Another essential point to be taken from this experiment is that the extension reaches an asymptotic plateau at almost half of the DNA contour length after Wi = 20.

**Fig. 6:**
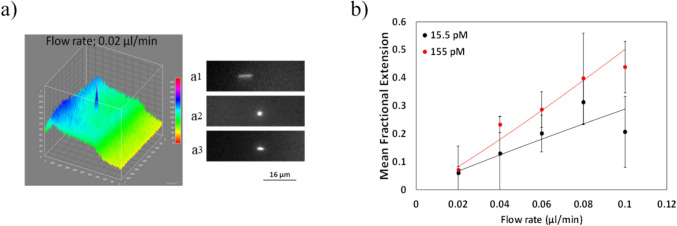
Fluorescence images of single λ-DNA molecules at different flow rates: **a**) 3D surface plot of a single DNA molecule at a flow rate of 0.02 µl/min (a_1_. a_2_. a_3_. Examples of a partially stretched single DNA molecule (0.02 µl/min)).** b**) Mean fractional extension versus flow rate and Wi for 2 different DNA concentration (e.g. 15.5 pM and 155 pM DNA)

In order to estimate the extension rate, single molecule image frames captured by the EMCCD camera [45–47] was used and acquired in ImageJ. Figure 5a shows a 3D surface plot of a single DNA molecule travelling in the microfluidic channel at a flow rate of 0.02 µl/min (Wi = 0.1). On the right hand side of the Fig. 6a, images are shown of DNA for repeat experiments using the same conditions. These images suggest that low flow rates used results in a relatively broad distribution in DNA conformation. The DNA end-to-end length is 7.3 µm in Fig. 6a1, while it is almost coiled up (measured length is 2.4 µm) in Fig. 6a2. Furthermore a partially stretched state with an extension length of 3.5 µm is shown in Fig. 6a3.

The correction factor is calculated from the distance that the fluid moves within the time period of the frame rate (30 ms) and subtracted from the measured DNA length. The mean fractional extension with the correction factor is plotted against flow rate in Fig. 6b. Mean fractional extension (MFE) is defined by;

10 $$MFE=\frac{\langle x\rangle }{{L}_{c}}$$

where $\langle x\rangle$ is the mean extension and L_c_ is the DNA contour length. At a flow rate of 0.1 µl/min (Wi = 0.38), MFE reaches a value of 0.21 and 0.43 for 15.5 pM and 155 pM DNA concentration, respectively.

This is in contrast to the findings reported by Smith et al.; ~ 0.4—0.5 at Wi < 20, and slightly higher than the literature. The discrepancy might arise from the differences in the experimental conditions such as buffer concentration. It is also important to remember that the position of the focal plane might be slightly off-centred. This would eventually cause deviations from the expectations due to the differences in the local shear rate, and thus in the elongation rate.

## Conclusion

In this study, flow velocity and DNA behaviour in flow was explored using confocal FM. Velocity measurements resulted in good agreement with the expected flow velocity. Confocal FM studies co-operated with FCS revealed the determination of diffusion- and flow-dominated regimes, which suggests that the system becomes flow-dominated at a flow rate of 0.3 µl/min. In wide field imaging experiments it was observed that DNA partially elongates to almost half of its counter length at a flow rate of 0.1 µl/min. However, the elongation rate varies across the channel because it is a dependant variable on the velocity and the local shear rate, which differ across the channel. Another experimental observation was that slower flow rates (e.g. 0.1 µl/min) caused a backlash in some experiments; this is perhaps due to the limitations of the pump and the friction of the tubing. This may have been a source of the differences in extension observed for repeats at low flow rates.

## Data Availability

No datasets were generated or analysed during the current study.
